# learnMSA2: deep protein multiple alignments with large language and hidden Markov models

**DOI:** 10.1093/bioinformatics/btae381

**Published:** 2024-09-04

**Authors:** Felix Becker, Mario Stanke

**Affiliations:** Institute of Mathematics and Computer Science, University of Greifswald, 17489 Greifswald, Germany; Institute of Mathematics and Computer Science, University of Greifswald, 17489 Greifswald, Germany

## Abstract

**Motivation:**

For the alignment of large numbers of protein sequences, tools are predominant that decide to align two residues using only simple prior knowledge, e.g. amino acid substitution matrices, and using only part of the available data. The accuracy of state-of-the-art programs declines with decreasing sequence identity and when increasingly large numbers of sequences are aligned. Recently, transformer-based deep-learning models started to harness the vast amount of protein sequence data, resulting in powerful pretrained language models with the main purpose of generating high-dimensional numerical representations, embeddings, for individual sites that agglomerate evolutionary, structural, and biophysical information.

**Results:**

We extend the traditional profile hidden Markov model so that it takes as inputs unaligned protein sequences and the corresponding embeddings. We fit the model with gradient descent using our existing differentiable hidden Markov layer. All sequences *and* their embeddings are jointly aligned to a model of the protein family. We report that our upgraded HMM-based aligner, learnMSA2, combined with the ProtT5-XL protein language model aligns on average almost 6% points more columns correctly than the best amino acid-based competitor and scales well with sequence number. The relative advantage of learnMSA2 over other programs tends to be greater when the sequence identity is lower and when the number of sequences is larger. Our results strengthen the evidence on the rich information contained in protein language models’ embeddings and their potential downstream impact on the field of bioinformatics.

**Availability and implementation:**  https://github.com/Gaius-Augustus/learnMSA, PyPI and Bioconda, evaluation: https://github.com/felbecker/snakeMSA

## 1 Introduction

An accurate model of sequence homology is crucial for transferring knowledge between related proteins and is important when inferring structure, function or phylogeny. Multiple sequence alignments (MSAs) are a widely used data structure to describe which sites of a set of sequences have evolved from a common ancestor by assigning them to columns. Modern aligners have to face a rapid scale-up in the number of input sequences (i.e. the alignment *depth*) as a consequence of data growth. For example, the Earth BioGenome project has the goal of sequencing about 1.8 million eukaryotic genomes within this decade (Lewin *et al.* 2022). The growth of protein family databases could outpace increases in CPU speed. Already, large superfamilies like ABC transporters encompass millions of sequences.

A common model for a protein family is the profile Hidden Markov Model (pHMM) (Baldi and Hunkapiller 1992, Krogh *et al.* 1994, Eddy 1995), which, in contrast to an MSA, provides a more concise description of homology. In particular, it is independent of *depth*. A pHMM consists of position-specific amino acid distributions that correspond to specific columns in an MSA along with additional information on insertions and deletions. MSAs and pHMMs are closely related, as each can be derived from the other. Commonly, pHMMs are fitted to aligned sequences (supervised). A pHMM can also be trained on *unaligned* sequences and an MSA can be inferred by aligning sequences to the pHMM. Although the latter approach to MSA has been noted before (Krogh *et al.* 1994, Eddy 1995), most popular modern aligners are *progressive*, i.e. they iteratively construct deeper MSAs from pairs of shallower MSAs. Only recently we have shown with our tool learnMSA that *unsupervised* pHMM training can be leveraged to construct MSAs with state-of-the-art accuracy when applied to large datasets (Becker and Stanke 2022). In this previous work, we trained a pHMM using gradient descent and an HMM layer as part of a deep-learning model. Note that learnMSA does not follow the usual paradigm of machine learning, where the parameters *θ* of a function $f_{\theta }$ are learned at training time and then used at inference time to map an input *x* to a prediction $f_{\theta }(x)$. Instead, learnMSA performs a training at inference time, when a user requests a set of proteins *x* to be aligned. The result of the (unsupervised) training are the parameters *θ* of the pHMM and the MSA is only a relatively minor postprocessing step. One major advantage of learnMSA is its ability to attend to *all* sequences when sorting residues into columns while in contrast for most other progressive and seed-based tools only parts of the data are used at a time. Yet another opportune feature is the fact that it does not require computing a tree over the input sequences, which is typically the computational bottleneck and a severe barrier when constructing very deep MSAs.

The unique approach of learnMSA is in stark contrast to other well-performing aligners suitable for large numbers of sequences (Santus *et al.* 2023). Most MSA methods have been developed over decades when moderately sized input sets dominated the research landscape. Many aligners are built on previously existing tools or methods. Still, MSA approaches are prevalent that break the problem down heuristically into multiple smaller alignment tasks. Progressive methods use a tree to guide stepwise pairwise alignments. Examples are Clustal Omega (Sievers *et al.* 2011), MAFFT (Katoh and Standley 2013), or the more recent high-throughput methods FAMSA (Deorowicz *et al.* 2016) and Kalign 3 (Lassmann 2020) that use hardware-close optimizations. Seed-based algorithms represent the input sequences by a subset, called the “seed,” that is aligned first. Examples are UPP (Nguyen *et al.* 2015, Park *et al.* 2023), which is based on pHMM ensembles and MAFFT-Sparsecore (Yamada *et al.* 2016), which aligns the seed sequences with a consistency-based algorithm. Cluster-based methods use a divide-and-conquer strategy, align smaller sets of sequences first, and subsequently merge them. Examples are MAGUS (Smirnov and Warnow 2021), the regressive variant of T-Coffee (Garriga *et al.* 2019), or MUSCLE5 (Edgar 2022). All approaches, progressive, cluster-, and seed-based, are heuristic and exploit only part of the data when making decisions about which sites are homologous. Erroneous decisions may accumulate during the progression of the heuristic and cannot be reversed except with costly iteration (Sievers *et al.* 2011, Garriga *et al.* 2019). For a more comprehensive overview of state-of-the-art large-scale aligners, we refer to a recent review (Santus *et al.* 2023).

Two issues with all modern aligners can be raised. First, all programs tend to be less accurate with a larger number of input sequences (Sievers *et al.* 2011, Garriga *et al.* 2019, Becker and Stanke 2022, Santus *et al.* 2023) (see Fig. 3b). A specific concern highlighted is the perhaps unexpected observation that MSA accuracy does not increase with data volume (Santus *et al.* 2023). The second challenge lies in the alignment of distantly related sequences with low sequence similarity. Evident relationships in the primary sequences may have diverged over the course of evolution and are hard to find if only amino acids are used as features.

### 1.1 Protein language modeling

The field of protein language models (pLMs) encompasses a broad range of variants of the transformer (Vaswani *et al.* 2017). Large language models have been applied to proteins with empirical success (Elnaggar *et al.* 2021, Brandes *et al.* 2022, Lin *et al.* 2022). Such models mostly differ in size and architectural details. For this work, we consider three established pLMs differing in the use of attention, number of parameters, positional embedding and pretraining objective. ESM-2 (Lin *et al.* 2022) is a family of modified BERT encoder models with up to 15 billion parameters. ProtT5-XL (Elnaggar *et al.* 2021) is a T5 encoder model with approximately 1.2 billion parameters. ProteinBERT is a smaller language model with only 16 *million* parameters, but it is fast and comparably lightweight and its pretraining objective is more tailored toward proteins (Brandes *et al.* 2022).

Embeddings output by pLMs unify a rich set of biological features like biophysical properties (Vig *et al.* 2020) or structural information (Alley *et al.* 2019, Vig *et al.* 2020, Rives *et al.* 2021). Moreover, simple linear models on top of the embeddings lead to accurate transfer predictions such as for binding sites (Vig *et al.* 2020) or residue–residue contacts (Rao *et al.* 2020, Vig *et al.* 2020). Embeddings can also encapsulate evolutionary information (Lin *et al.* 2022), historically obtained from MSAs, when the pLM is trained on unaligned proteins.

### 1.2 Related work

Recently, embedding-based *pairwise* sequence alignment algorithms have been introduced that use the Smith–Waterman dynamic programming scheme. These methods construct context-sensitive scoring matrices by pairing all residue embeddings for two sequences using bilinear symmetric forms (Llinares-López *et al.* 2023), cosine similarity (Kaminski *et al.* 2023), or standardized Euclidean distances (Pantolini *et al.* 2024). All approaches report significantly higher accuracy over existing methods on very dissimilar sequences. For pairwise alignment, incorporating pLMs yields a clear advantage over traditional fixed and context-independent amino acid scoring matrices.

Using embeddings for *multiple* alignment is currently still in its infancy. While the Smith–Waterman algorithm for aligning two sequences considers all pairs *i*_1_, *i*_2_ of sequence indices for possible alignment columns it is computationally infeasible to consider all tuples $i_{1},\ldots ,i_{n}$ when aligning n≫2 sequences. Certainly, similar heuristics are possible to those for amino acid scoring schemes. Those could break down the task of embedding-based multiple alignments into a sequence of pairwise alignment tasks. However, such approaches could suffer from the same algorithmic disadvantages of accumulating errors in a large number of sequentially made alignment decisions. It might be beneficial to formulate the alignment as a joint optimization problem depending end-to-end on *all* input sequences and their embeddings. Petti *et al.* introduced a simple generalization of the differentiable Smith–Waterman algorithm that constructs an MSA with end-to-end learning by aligning all sequences to one reference (Petti *et al.* 2023). However, Petti *et al.* do not use an HMM to define a distribution of alignments, insertions relative to the reference sequence remain unaligned and no standalone MSA tool is provided. McWhite *et al.* used embeddings as static features in an algorithm to cluster and order columns (McWhite *et al.* 2023). This approach also reports higher alignment accuracy in the presence of low sequence similarity; however, it is currently limited to low numbers of sequences.

## 2 Materials and methods

In this work, we present an extension of the pHMM class that is commonly used to model protein families [e.g. in Pfam (Mistry *et al.* 2021)] and for sensitive homology searches [HMMER (Mistry *et al.* 2013), HHsearch (Söding 2005)]. Our goal was to enable profile models to leverage features that come as embeddings, i.e. high-dimensional numerical vectors. Currently, our focus is on training the extended models on large datasets of *unaligned* sequences and consequently decoding from those models more accurate alignments compared to state-of-the-art tools (including our own pHMM-based aligner learnMSA) that use only amino acids and very limited prior knowledge, such as scoring matrices or Dirichlet distributions.

A pHMM describes a family of protein sequences and its evolution with a stochastic process over latent (hidden) discrete variables and observed variables that represent an unaligned protein sequence. The pHMM has position-specific amino acid distributions and accounts for the occurrence of insertions and deletions and, in some cases, flanking segments, domain repeats, or fragmentation (Eddy 2008). The marginal probability distribution over protein sequences is obtained by summing over *all* possible alignments of a sequence to the model. This assigns high probabilities to family members and low probabilities to other proteins.

Recently, we have introduced a unique approach to the multiple alignment task. Our tool learnMSA implements a differentiable pHMM (Becker and Stanke 2022). learnMSA learns HMM- and other parameters jointly via batch gradient descent by maximizing an objective that combines the likelihood of the unaligned input sequences and a prior. Supplementary Fig. S6 and Supplementary Video S7 show the amino acid profile built at the end or after each training step, respectively, as learnMSA iterates over input set of unaligned sequences. The initial distribution is determined by a prior and has a large entropy and low information. The profile gradually concretizes over the course of the optimization. The method presented here is a direct improvement of the original learnMSA.

### 2.1 Deep profile hidden Markov models

The likelihood of a sequence S given an HMM *θ* is a sum over all possible alignments, each represented by a path of states *π*: $P_{\theta }(S)=\sum_{\pi }P_{\theta }(\pi ,S)=\sum_{\pi }\prod_{j}P_{\theta }(\pi_{j}|\pi_{j-1})P_{\theta }(S_{j}|\pi_{j})$. Here, *π_j_* is the hidden state corresponding to $S_{j}$. $P_{\theta }(S)$ can be efficiently computed with either the forward or backward algorithm. Both algorithms can be implemented in a fully differentiable and vectorized variant (Becker and Stanke 2022). In order to incorporate embedding vectors, we will modify only the emission probabilities $P_{\theta }(S_{j}\;|\;q)$ of the pHMM used in learnMSA where *q* denotes any state of the model.

If S is a protein sequence and ϕ a protein language model (pLM), we call the *L *×* d* matrix ϕ(S) embedding of S of dimension *d* (a hyperparameter of ϕ). Let $\phi {\left(S\right)}_{j}$ denote the vector embedding corresponding to residue $S_{j}$ that was computed specifically for the site and that captures bidirectional context. We define a pHMM with the concatenated input (S,ϕ(S)) as described below.

We assume conditional independence of amino acid and embedding and further introduce a temperature parameter *τ* to regulate the relative influence of embeddings:

(1) $$P(S_{j},\phi {\left(S\right)}_{j}|q)=P(S_{j}|q)P{\left(\phi {\left(S\right)}_{j}|q\right)}^{\tau }.$$

**Figure 1.**
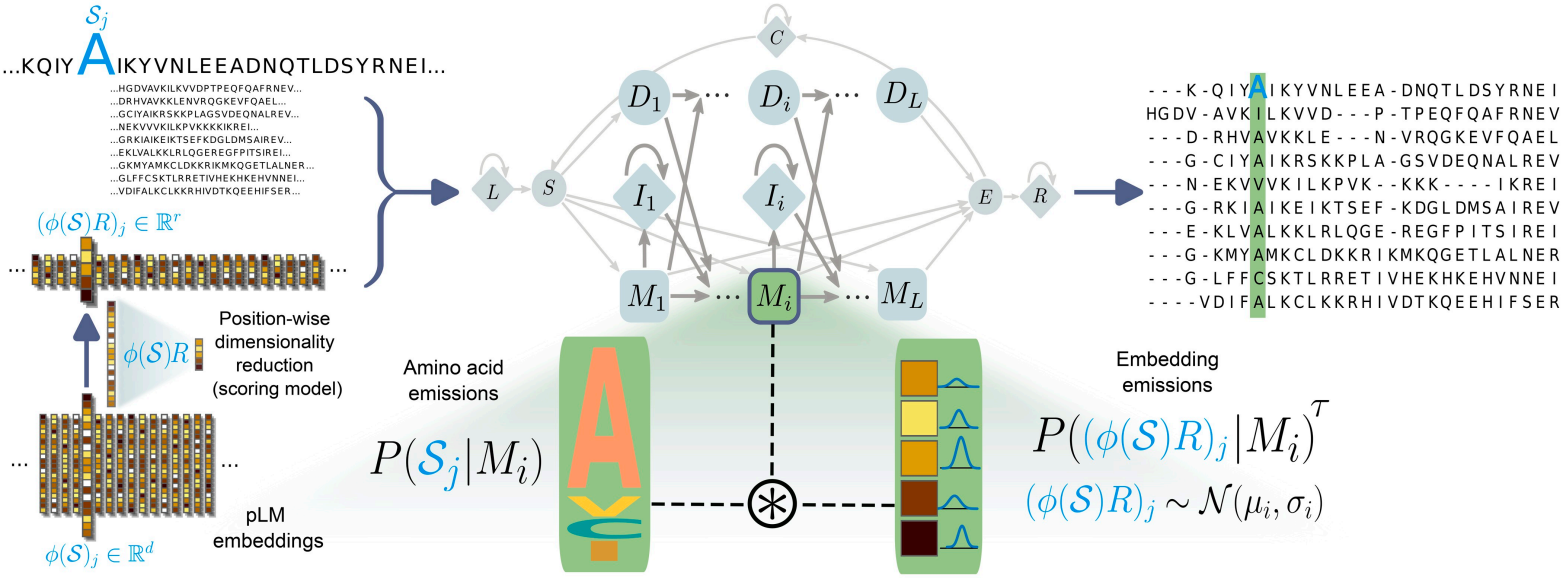
All-to-one alignment of sequences and their embeddings to an extended profile hidden Markov model. The pLM that embeds sequences is denoted by ϕ. Match states encompass a joint model for emissions of amino acids $S_{j}$ and embedding vectors ${\left(\phi (S)R\right)}_{j}$. The matrix *R* is pretrained in advance.

A deep profile Hidden Markov Model (dpHMM) therefore describes a protein family by modeling the *joint* distribution of the amino acid sequence and the embedded sequence (also see Fig. 1). For each conserved site of the family, a distribution over amino acids and a distribution over embeddings is parametrized, that together yield an amino acid profile and a *profile of embeddings*.

As a probabilistic model of embeddings at a match state *q* = *M_i_* we assume $\phi {\left(S\right)}_{j}∼N(\mu_{i},\sigma_{i})$ where *μ_i_* and *σ_i_* are learned parameters associated with state *M_i_* and N(μ,σ) is the multivariate normal distribution with mean vector *μ* and covariance matrix *σ*. We chose *σ_i_* to be a diagonal matrix. Consequently, *σ_i_* contains the variances of each embedding dimension and *μ_i_* is the expected embedding vector at state *M_i_*. We chose $P(\phi {\left(S\right)}_{j}|q)$ to be the density function of the described multivariate normal distribution. We considered more complex alternatives such as full covariance matrices or a mixture of Gaussians, but found them to be computationally infeasible. The parameters *μ_i_* and *σ_i_* are learned jointly with the remaining HMM parameters using gradient descent. We discuss the initialization of *μ* and *σ*, the choice of the temperature *τ* and how we handle insertions in the Supplementary Material.

A dpHMM has many potentials over a traditional pHMM. The embedding of a residue depends on its context, e.g. whether its neighborhood is hydrophobic. In principle, even interactions of residues that are widely spaced in the primary sequence (but proximate in 3D space) could have been implicitly learned by the pLM and be a criterion in the alignment. For example, structural information contained in the embeddings can be exploited, as has been shown for ProtT5-XL embeddings, which can be used to accurately predict secondary structure (Elnaggar *et al.* 2021). Single neurons in embeddings have been found to correlate with secondary structure elements (Alley *et al.* 2019) and it has been shown that attention scores (which are based on embeddings) align closely with contact maps (Vig *et al.* 2020). In contrast, long-range dependencies are not captured by a traditional pHMM where amino acid emissions are conditionally independent given a state. The underlying Markov chain simplifies the true evolutionary process and does not account for long-range interactions of sites through its architecture.

### 2.2 Scoring model

So far, we have described a general dpHMM that uses any kind of numeric vector inputs of dimension *d*. We found that there are two critical problems that potentially hinder an application of dpHMMs on embeddings as *direct* output of a pLM. As a consequence of the high dimensionality of embeddings (i.e. *d* large), the number of dpHMM parameters will greatly increase with the complexity of the language model. This poses the risk of overfitting, in particular when the number of sequences to be aligned is limited and has severe downsides from a computational perspective. Furthermore, typically language model embeddings are not specialized for the MSA task as they were never trained on aligned sequences. Thus, they can be expected to contain many features not directly relevant to MSA or sequence family modeling. To solve these issues, we insert an intermediate model between pLM and dpHMM which we call the *scoring model*.

**Figure 2.**
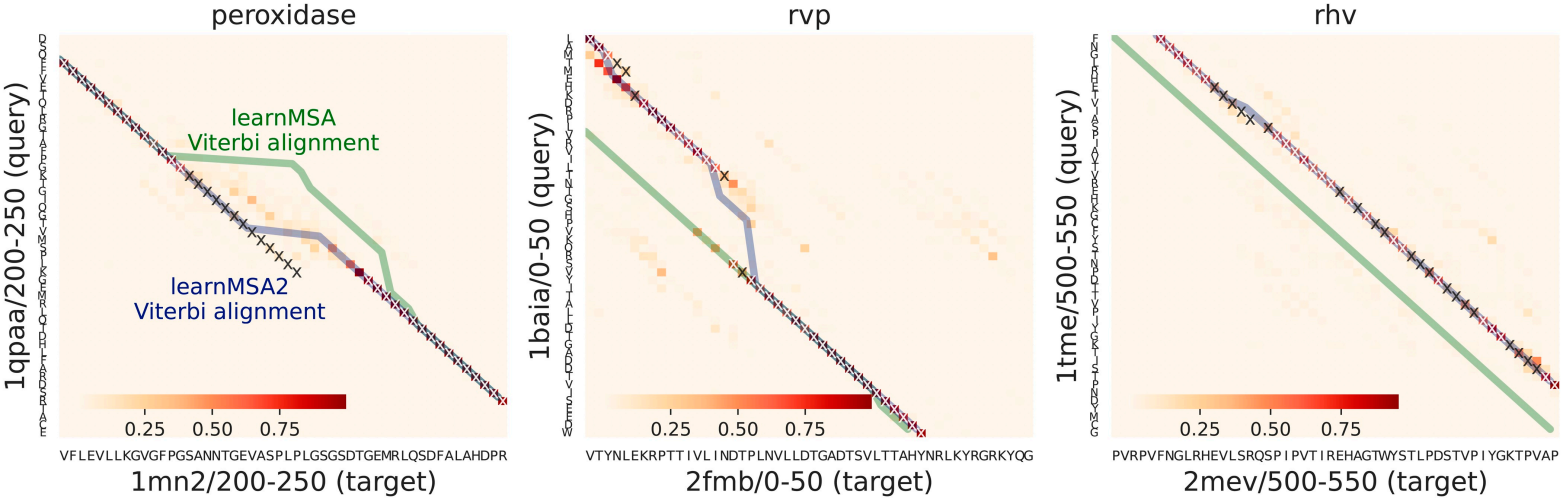
Residue–residue alignment probabilities *A* (yellow to red palette) based on ProtT5-XL. The true alignment is marked by ×. The blue (green) path shows learnMSA’s alignment with (without) support of *A.*

The scoring model *ζ* has two modes: During **inference**, it serves as a compression of embeddings into a simpler space $\zeta :R^{d}\to R^{r}$ with r≪d. This mode is used when training a dpHMM (where the parameters of *ζ* are frozen) and when decoding an MSA. During (pre)**training** of *ζ*, the scoring model takes a pair (ϕ(S),ϕ(T)) of embedded sequences as input and maps them to a probabilistic matrix $\left(A_{i,j}\right)$, where $A_{i,j}$ depends on $\phi {\left(S\right)}_{i}$ and $\phi {\left(T\right)}_{j}$ and scores how well the positions *i* and *j* align (see Fig. 2). *ζ* is pretrained on a large collection of “correct” pairwise alignments inferred from MSAs, i.e. the cross-entropy between $A_{i,j}$ and a “correct” reference is minimized.

A pair of vector embeddings $\phi {\left(S\right)}_{i},\phi {\left(T\right)}_{j}\in R^{d}$, column-vectors corresponding to the *i*th and *j*th residues of two different sequences, will be scored using a symmetric bilinear form $\phi {\left(S\right)}_{i}^{T}W\phi {\left(T\right)}_{j}$ where $W\in R^{d\times d}$ is a symmetric matrix. This score is contextual and this approach has been used before for pairwise alignment (Llinares-López *et al.* 2023). To allow embedding compression and to reduce the number of parameters of the scoring model and consequently overfitting, we use a *low-rank* parametrization of *W*, similar to low-rank adaption commonly used for transformer fine-tuning (Hu *et al.* 2022): We set $W:=RR^{T}$ where $R\in R^{d\times r}$ with r≪d. As default, we chose *r *=* *16 but also experimented with larger values.

Therefore, we define the len(S)×len(T) matrix of scores as

(2) $$A:=f\left(\phi \left(S\right)R{\left(\phi \left(T\right)R\right)}^{T}\right),$$

where *f* is a differentiable activation function, which we chose to be the logistic sigmoid function. In this case, the pretraining task of the scoring model can be formulated as the binary classification problem of independently predicting the probability of residue–residue alignment for all residue pairs. For training, we used the cross entropy loss function with supervision from pairwise alignments deemed correct. *A* can also be interpreted as the matrix of cross-attention scores, (as similarly used in transformers) between S and T.

During inference of *ζ*, i.e. when compressing embeddings, we compute:

(3) ζ(ϕ(S))=ϕ(S)R.

Note that we pretrain the scoring model *ζ* to be family agnostic. Thus, we will pretrain a scoring model *ζ* once and use it with frozen parameters when learning a protein family and constructing an MSA. When pretraining *ζ*, gradients are computed only with respect to *ζ*, the parameters of ϕ were frozen. Thus, *ζ* is compatible with the original parameter set published for the respective pLMs and we only have to distribute a very small set of additional parameters. Our data pipeline for pretraining the scoring model is described later.

A set of possible pairwise alignments can be interpreted as alternative paths in a grid with the sequences as axes as seen in Fig. 2. When inferring a concrete alignment, we consider the path with maximum probability (or score) among all possible paths. When training a dpHMM, all possible alignments are considered in an efficient manner, eliminating the need for hard decisions in the presence of uncertainties in the alignment space—especially when sequences are only distantly related. Thus, training a dpHMM on unaligned sequences considers any alignment of any sequence at all times and only their probabilities vary during the learning phase. In this context, the incorporation of embedding information output by a pLM can yield evidence toward specific alignments.

### 2.3 Additional details

We assume a mixture of multivariate normal distributions as a prior over the embedding emission distributions, i.e. for all match states *M_i_* we let $\mu_{i}∼\sum_{c=1}^{C}\pi_{c}N(\mu_{c},\sigma_{c})$, where *σ_c_* is again a diagonal matrix. The embedding prior is an analog to the mixture of Dirichlet distributions typically assumed for amino acid distributions in profile models. It is pretrained on embeddings of residues found in densely populated alignment columns in order to ensure that *μ_i_* resembles actual embeddings generated by the pLM.

We implemented a modular framework that allows the addition of a new embedding distribution, including a prior distribution for it, to our existing training and alignment pipeline. When running learnMSA2 with language models (--use_language_model) we only train embedding emission distributions at the match states (--frozen_insertions). The reduced embeddings (Equation 3) are precomputed and cached for all sequences before training. For reference, storing the 32 bit embeddings of the largest family during our experiments, ABC transporters, with about 3 million sequences compressed to *r *=* *16, takes about 35 GB RAM.

## 3 Evaluation and discussion

### 3.1 MSA benchmarking data and tools

To evaluate the accuracy of MSAs, we used the HomFam collection (Sievers *et al.* 2011). Each family in HomFam has a subset of sequences (*reference set*) with a gold standard alignment. The remaining protein sequences form a large set of presumable homologs collected from Pfam. HomFam contains 94 protein families that encompass a wide spectrum of identity levels (17.8%–76.5%), dataset depths (93–93681 sequences), and sequence lengths (12–854). We also evaluated on extHomFam (Deorowicz *et al.* 2016) that extends HomFam with additional families and additional homologs (up to 3M sequences).

We use the reference-based sum-of-pairs (SP), total column (TC), and column scores, which we calculated using T-Coffee with the option aln_compare. SP (sum-of-pairs) score is the percentage of residue pairs in the reference that are correctly aligned. TC (total column) score is the percentage of alignment columns in the reference that have a matching column in the prediction. The column score is a weighted variant of the TC score that weights each column with the number of residue–residue pairs in the column divided by the total number of pairs in all columns. Thus, the column score considers dense columns of the reference alignment more important and is more forgiving when aligning sparse columns incorrectly.

We tested six established aligners for large numbers of sequences: Clustal Omega (Sievers *et al.* 2011), MAFFT-Sparsecore (Yamada *et al.* 2016), MUSCLE5 (Edgar 2022), T-Coffee (regressive) (Garriga *et al.* 2019), FAMSA (Deorowicz *et al.* 2016), and MAGUS (Smirnov and Warnow 2021) (see Supplementary Material for versions and command lines). Note that none of the competing aligners is based on deep learning or uses pLM embeddings. To our knowledge, there is no other aligner that uses pLM and is suitable to align a large number of sequences.

### 3.2 Pretraining data

We used aligned protein sequences to pretrain the scoring model and the prior embedding distribution. For this purpose, we used Pfam (Mistry *et al.* 2021) seed alignments. From those multiple alignments, we induced pairwise alignments. We excluded any Pfam family that hits at least one sequence when searching against the HomFam collection. For this, we used MMSeqs2 (Steinegger and Söding 2017) with an identity threshold of 50% (see Supplementary Material for details). By removing full families, we also excluded training sequences with less than 50% similarity to test sequences and computed an out-of-family error on the test data. Note that the scoring model and the prior contain relatively few parameters (16 384 and 1040, respectively) compared to the amount of training data and pose very little risk of overfitting. Also note that the language model itself has most likely seen examples from the families contained in the HomFam collection during its unsupervised pretraining; however, not their alignments.

### 3.3 Embeddings improve MSA accuracy

**Figure 3.**
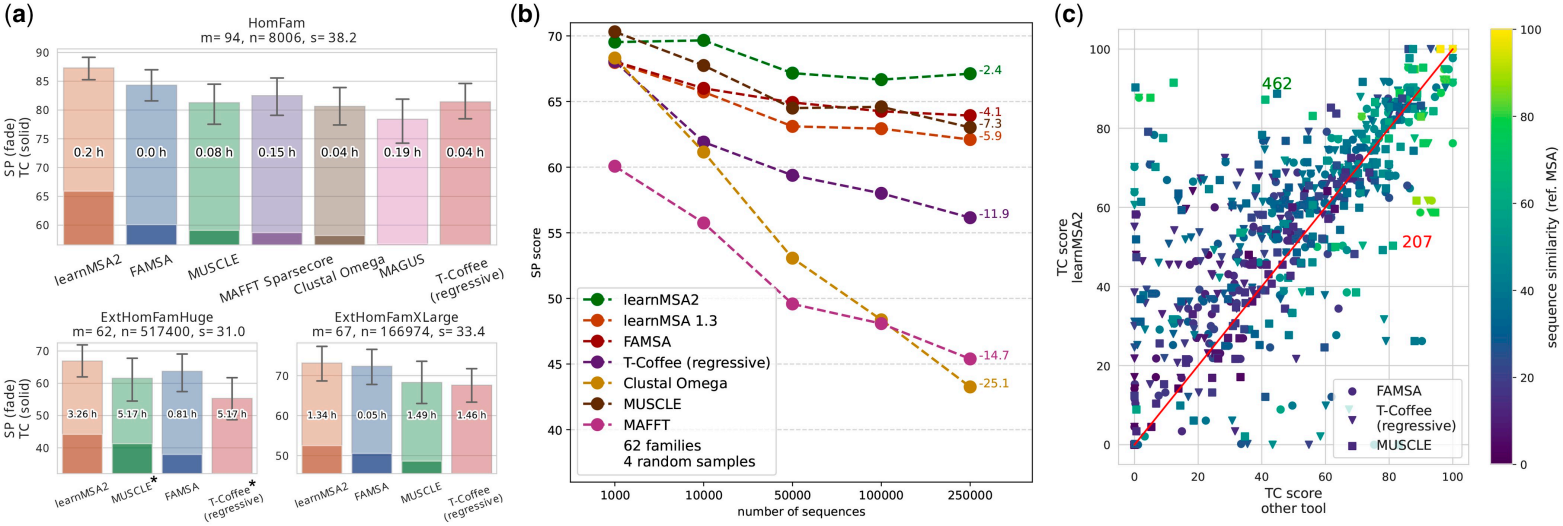
(a) Accuracy comparison of learnMSA2 to other aligners. SP (sum-of-pairs) score and TC (total column) score are defined in the text. The bars are sorted by TC score. The each bar is labeled with the average runtime in hours per MSA rounded to two decimals. The *y* axis starts at the lowest average TC score achieved by any tool on the respective dataset. 95% confidence intervals are plotted for SP scores (whisker). *m*: number of protein families, *n*: average number of sequences (depth), *s*: average sequence similarity in %, (asterisk): MUSCLE (T-Coffee at tree building stage) failed 1 (5) MSAs and were evaluated only on the subset of families they successfully aligned. (b) Alignment SP score as a function of the number of input proteins evaluated on the same references embedded into increasingly large, nested sets of homologs (more details given in text). The rightmost datapoints are labeled with the score difference of the last and the first datapoint. (c) Relative TC score comparison of learnMSA2 and selected other methods. Each datapoint represents a pair of MSAs performed on the same set of proteins. Sequence similarity and improvements in alignment accuracy (*y–x*) correlate negatively (ρ=−0.14). The number in the upper (lower) triangle shows the number of MSAs learnMSA2 aligned better (worse).

learnMSA2 is more accurate than established tools that only use amino acids (Fig. 3a) and the previous versions of learnMSA, that do not use language model embeddings (see Supplementary Fig. S1). Its alignment accuracy relative to other tools increases when the pairwise similarity of the sequences decreases (see Fig. 3c, Pearson correlation of similarity and score difference to FAMSA ρ=−0.15, T-Coffee ρ=−0.27, and MUSCLE ρ=−0.01). This effect is less prominent in the case of MUSCLE, which uses the consistency-based tool ProbCons. At the same time, the accuracy of learnMSA2 increases—as expected—with the similarity of the proteins that ought to be aligned (ρ=0.49). We conclude that the relative advantages of learnMSA2 are greatest for dissimilar and difficult-to-align families. Moreover, the average relative accuracy of learnMSA2 tends to increase the deeper the input alignment is (Fig. 3b, Supplementary Fig. S3).

In all experiments, we observed that the positive effect of pLM embeddings on the TC score was disproportionally larger than on the SP score and, furthermore, on column score (see Supplementary Fig. S2) disproportionally larger than on the TC score. Therefore, although beneficial in all respects, the use of embeddings seems more helpful to construct correct alignment *columns* rather than correct residue pairs. Also, it tends to help more when weighting dense columns up, which are putatively more structurally or evolutionary relevant. This agrees with our initial motivation to improve alignment with evidence based on biophysical and structural properties.

### 3.4 Runtime and time complexity

It is noteworthy that, unlike established tools, learnMSA2’s runtime is in $O(nL^{3})$, where *L* is the sequence length, and thus it scales linearly with the number of input sequences *n*, since it does not require computing a tree. Indeed, the runtime ratio of learnMSA2 to other methods, even to high-throughput tools such as FAMSA, decreases when aligning up to millions of sequences as seen in Fig. 3a. The cubic scaling in *L* is of limited impact because protein lengths are bounded in practice. See Supplementary Figs S4 and S5 for a detailed runtime comparison.

### 3.5 Choice of the language model and hyperparameter search

We evaluated our method with 3 different pLMs: ESM-2 in the second largest variant (2.8B parameters, *d *=* *2560), ProtT5-XL-UniRef50 (1.2B parameters, *d *=* *1024), and ProteinBERT (16M parameters, *d *=* *1562). ProteinBERT has substantially fewer parameters but a large embedding size because it concatenates the outputs of intermediate layers. We also varied the hyperparameters of the scoring model r∈{16,32,64} and C∈{1,10,32}. When grouped by language model, learnMSA2 with ProtT5-XL embeddings aligned HomFam on average with 66.00 (87.30) TC (SP) score, while with ESM-2 it achieved 65.14 (86.67) and with ProteinBERT 64.02 (85.88). The (by a large margin) smallest model, ProteinBERT, did only achieve a minor gain of 0.9 (0.7) in TC (SP) score over learnMSA2 without embeddings. We found that with small *r* learnMSA2 was not less accurate than with larger *r* while being substantially faster and more memory-friendly. Moreover, the choice of C≥1 has little impact on the results. We chose *r *=* *16 with sigmoid activation and *C *=* *32 as the default values for learnMSA2.

### 3.6 Data harms alignment accuracy

We carried out a large-scale experiment to study alignment accuracy as a function of the number of input sequences (see Fig. 3b). For this we took the 62 largest datasets from the extHomFam collection with at least 250K proteins per family. We created nested subsamples with the following procedure, which reversely simulates the situation where a protein family alignment shall be constructed and more and more family members become available over time. First, the reference sequences of each family were embedded in four randomly sampled subsets of the available homologs. This resulted in 4 × 62 datasets with exactly 250K sequences each. Second, each dataset was further sampled down in sequences by removing random homologs until a depth of 100K, 50K, 10K, and 1K was reached. The reference sequences were kept at all times. Third, all datasets were aligned with different tools and the induced MSAs of the reference sequences were evaluated.

Contrary to the intuition that additional data should rather help the correct alignment of the reference sequences, the accuracy of the subalignment of reference sequences declines for all methods when the number of additional sequences grows large (Fig. 3b). learnMSA2 has the lowest accuracy decline among the methods tested although the problem is still noticeable. As learnMSA2 is somewhat less impacted by a depth increase than learnMSA without pLM, we conclude that the addition of embeddings has the tendency to stabilize alignment accuracy when the number of input proteins increases. However, none of the tested tools was able to increase accuracy with more available data.

### 3.7 Hardware requirements

Large-scale experiments were performed on a cluster with 250 GB of memory and 32 CPU cores allocated per MSA job. learnMSA was the only tool that could compute MSAs fully using GPU resources. We used NVIDIA A100 GPUs (80 GB). However, learnMSA2 also runs on smaller scale hardware like NVIDIA RTX 4090 GPUs (24 GB) and in principle on even smaller GPUs, if the batch size or the number of HMMs trained in parallel is reduced.

Running learnMSA2 on CPU is possible, however, if pLMs embeddings are used, it aligns HomFam 15 times slower (3 h on average per family) than learnMSA2 utilizing GPU (0.2 h) and 16 times slower than the slowest CPU method (MAGUS, 0.19 h). When running learnMSA2, enabling pLM support roughly doubles the (GPU) memory requirements.

It is necessary to download the original weights of a language model when using learnMSA2. Our default choice, ProtT5-XL in the encoder-only, half-precision variant, requires roughly 2.5 GB of disk space. The model is automatically downloaded when first running learnMSA2 with the option --use_language_model.

### 3.8 Current limitations of learnMSA

To learn an accurate model, learnMSA requires a minimum number of sequences (in most cases starting at 1000, a few hundred might still be enough) to achieve state-of-the-art accuracy. Aligning very small numbers of sequences with learnMSA leads to very poor alignments. We recommend using an established alternative for such shallow MSAs. Currently, learnMSA supports only protein alignments. We may extend it in the future to align DNA or RNA sequences.

## 4 Conclusion

We show that residue-level embeddings from large protein language models can be leveraged to increase alignment accuracy. The accuracy advantage of embedding-based over amino acid-based MSAs increases with increasing alignment depth (number of input sequences) and decreasing sequence similarity. Our results confirm and extend the potential of pLMs in spearheading a new era of algorithms that leverage the enriched feature space of sequence embeddings. The choice of language model matters and it is not an exclusive question of sheer model size: We found ProtT5-XL to yield the most accurate MSAs despite being not the largest model indicating that model architecture or training approaches are important factors.

Our tool learnMSA does not require a guide tree. The properties and construction algorithms of guide trees and their impact on MSA are debated in Boyce *et al.* (2014), Deorowicz *et al.* (2016), Santus *et al.* (2023). It is surprising that tree-free alignment can surpass state-of-the-art accuracy on deep datasets.

Hitherto, learnMSA2 remains the only scalable program that can learn a pHMM for a protein family *without requiring or building an MSA*. Therefore, the remote homology search with a pHMM for new members of a given protein family does not necessarily require anymore to align the family. Since learnMSA2 improves alignment accuracy, we assume that the learned HMMs could lead to more accurate homology classification as well. Moreover, learnMSA2 can output *probabilistic* MSAs and is suitable for end-to-end learning of downstream tasks that can take an alignment as input (e.g. structure prediction). In such a case, the HMM as model for a distribution of alignments can be trained jointly with the downstream task.

We think that language modeling for biological sequences carries an immense potential still to be uncovered in many areas. This novel and powerful feature space can be combined with established models and algorithms to unlock its full potential.

## Supplementary Material

btae381_Supplementary_Data

## References

[btae381-B1] Aley EC , Khimulya G, Biswas S et al Unified rational protein engineering with sequence-based deep representation learning. Nat Methods 2019;16:1315–22. 31636460 10.1038/s41592-019-0598-1 PMC7067682

[btae381-B2] Baldi P , Hunkapiller T. Hidden Markov models in molecular biology: new algorithms and applications. Adv Neural Inf Process Syst 1992;5:747–54.

[btae381-B3] Becker F , Stanke M. learnMSA: learning and aligning large protein families. GigaScience 2022;11:giac104. 36399060 10.1093/gigascience/giac104 PMC9673500

[btae381-B4] Becker K , Sievers F, Higgins DG. Simple chained guide trees give high-quality protein multiple sequence alignments. Proc Natl Acad Sci USA 2014;111:10556–61. 25002495 10.1073/pnas.1405628111 PMC4115562

[btae381-B5] Brandes N , Ofer D, Peleg Y et al ProteinBERT: a universal deep-learning model of protein sequence and function. Bioinformatics 2022;38:2102–10. 35020807 10.1093/bioinformatics/btac020 PMC9386727

[btae381-B6] Deorowicz S , Debudaj-Grabysz A, Gudyś A. FAMSA: fast and accurate multiple sequence alignment of huge protein families. Sci Rep 2016;6:33964. 27670777 10.1038/srep33964 PMC5037421

[btae381-B7] Eddy SR. A probabilistic model of local sequence alignment that simplifies statistical significance estimation. PLoS Comput Biol 2008;4:e1000069. 18516236 10.1371/journal.pcbi.1000069 PMC2396288

[btae381-B8] Eddy SR. Multiple alignment using hidden Markov models. ISMB 1995;3:114–20. 7584426

[btae381-B9] Edgar RC. Muscle5: high-accuracy alignment ensembles enable unbiased assessments of sequence homology and phylogeny. Nat Commun 2022;13:6968. 36379955 10.1038/s41467-022-34630-w PMC9664440

[btae381-B10] Elnaggar A , Heinzinger M, Dallago C et al Prottrans: toward understanding the language of life through self-supervised learning. IEEE Trans Pattern Anal Mach Intell 2021;44:7112–27. 10.1109/TPAMI.2021.3095381 34232869

[btae381-B11] Garriga E , Di Tommaso P, Magis C et al Large multiple sequence alignments with a root-to-leaf regressive method. Nat Biotechnol 2019;37:1466–70. 31792410 10.1038/s41587-019-0333-6 PMC6894943

[btae381-B12] Hu EJ , Shen Y, Wallis P et al LoRA: low-rank adaptation of large language models. International Conference on Learning Representations 2022; https://openreview.net/forum?id=nZeVKeeFYf9.

[btae381-B13] Kaminski K , Ludwiczak J, Pawlicki K et al pLM-BLAST: distant homology detection based on direct comparison of sequence representations from protein language models. Bioinformatics 2023;39:btad579. 37725369 10.1093/bioinformatics/btad579 PMC10576641

[btae381-B14] Katoh K , Standley DM. MAFFT multiple sequence alignment software version 7: improvements in performance and usability. Mol Biol Evol 2013;30:772–80. 23329690 10.1093/molbev/mst010 PMC3603318

[btae381-B15] Krogh A , Brown M, Mian IS et al Hidden Markov models in computational biology: applications to protein modeling. J Mol Biol 1994;235:1501–31. 8107089 10.1006/jmbi.1994.1104

[btae381-B16] Lassmann T. *Kalign 3: Multiple Sequence Alignment of Large Datasets*. Bioinformatics 2019;36:1928–29. 10.1093/bioinformatics/btz795 PMC7703769 31665271

[btae381-B17] Lewin AH , Richards S, Aiden EL et al *The Earth BioGenome Project 2020: Starting the Clock*. Proceedings of the National Academy of Sciences 2022;119:e2115635118. 10.1073/pnas.2115635118 PMC8795548 35042800

[btae381-B18] Lin Z , Akin H, Rao R et al Evolutionary-scale prediction of atomic-level protein structure with a language model. Science 2023;379:1123–30. 36927031 10.1126/science.ade2574

[btae381-B19] Llinares-López F , Berthet Q, Blondel M et al Deep embedding and alignment of protein sequences. Nat Methods 2023;20:104–11. 36522501 10.1038/s41592-022-01700-2

[btae381-B20] McWhite CD , Armour-Grab I, Singh M et al Leveraging protein language models for accurate multiple sequence alignments. Genome Res 2023;33:277675. 10.1101/gr.277675.123 PMC10538487 37414576

[btae381-B21] Mistry J , Finn RD, Eddy SR, et al Challenges in homology search: HMMER3 and convergent evolution of coiled-coil regions. Nucleic Acids Res 2013;41:e121. 23598997 10.1093/nar/gkt263 PMC3695513

[btae381-B22] Mistry J , Chuguransky S, Williams L, et al Pfam: the protein families database in 2021. Nucleic Acids Res 2021;49:D412–9. 33125078 10.1093/nar/gkaa913 PMC7779014

[btae381-B23] Nguyen N-PD , Mirarab S, Kumar K et al Ultra-large alignments using phylogeny-aware profiles. Genome Biol 2015;16:124–15. 26076734 10.1186/s13059-015-0688-z PMC4492008

[btae381-B24] Pantolini L , Studer G, Pereira J et al Embedding-based alignment: combining protein language models with dynamic programming alignment to detect structural similarities in the twilight-zone. Bioinformatics 2024;40:btad786. 10.1093/bioinformatics/btad786 PMC10792726 38175775

[btae381-B25] Park M , Ivanovic S, Chu G et al UPP2: fast and accurate alignment of datasets with fragmentary sequences. Bioinformatics 2023;39:btad007. 36625535 10.1093/bioinformatics/btad007 PMC9846425

[btae381-B26] Petti S , Bhattacharya N, Rao R et al End-to-end learning of multiple sequence alignments with differentiable Smith–Waterman. Bioinformatics 2023;39:btac724. 36355460 10.1093/bioinformatics/btac724 PMC9805565

[btae381-B27] Rao R , Meier J, Sercu T et al Transformer protein language models are unsupervised structure learners. bioRxiv, 2020, preprint: not peer reviewed.

[btae381-B28] Rives A , Meier J, Sercu T et al Biological structure and function emerge from scaling unsupervised learning to 250 million protein sequences. Proc Natl Acad Sci USA 2021;118:e2016239118. 33876751 10.1073/pnas.2016239118 PMC8053943

[btae381-B29] Santus L , Garriga E, Deorowicz S et al Towards the accurate alignment of over a million protein sequences: current state of the art. Curr Opin Struct Biol 2023;80:102577. 37012200 10.1016/j.sbi.2023.102577

[btae381-B30] Sievers F , Wilm A, Dineen D et al Fast, scalable generation of high-quality protein multiple sequence alignments using Clustal Omega. Mol Syst Biol 2011;7:539. 21988835 10.1038/msb.2011.75 PMC3261699

[btae381-B31] Smirnov V , Warnow T. MAGUS: multiple sequence alignment using graph clustering. Bioinformatics 2021;37:1666–72. 33252662 10.1093/bioinformatics/btaa992 PMC8289385

[btae381-B32] Söding J. Protein homology detection by HMM–HMM comparison. Bioinformatics 2005;21:951–60. 15531603 10.1093/bioinformatics/bti125

[btae381-B33] Steinegger M , Söding J. MMseqs2 enables sensitive protein sequence searching for the analysis of massive data sets. Nat Biotechnol 2017;35:1026–8. 29035372 10.1038/nbt.3988

[btae381-B34] Vaswani A , Shazeer N, Parmar N et al Attention is all you need. Adv Neural Inf Process Syst 2017;30:5998–08.

[btae381-B35] Vig J , Madani A, Varshney LR et al BERTology meets biology: interpreting attention in protein language models. arXiv, arXiv:2006.15222, 2020, preprint: not peer reviewed.

[btae381-B36] Yamada KD , Tomii K, Katoh K et al Application of the MAFFT sequence alignment program to large data–reexamination of the usefulness of chained guide trees. Bioinformatics 2016;32:3246–51. 27378296 10.1093/bioinformatics/btw412 PMC5079479

